# Silencing the gustatory receptor BtGR11 affects the sensing of sucrose in the whitefly *Bemisia tabaci*

**DOI:** 10.3389/fbioe.2022.1054943

**Published:** 2022-11-14

**Authors:** Fengqi Li, Zhongjuan Di, Jiahui Tian, Youssef Dewer, Cheng Qu, Shiyong Yang, Chen Luo

**Affiliations:** ^1^ Beijing Key Laboratory of Environment Friendly Management on Fruit Diseases and Pests in North China, Institute of Plant Protection, Beijing Academy of Agriculture and Forestry Sciences, Beijing, China; ^2^ Key Laboratory of Chemical Biology and Molecular Engineering of Ministry of Education, Institute of Biotechnology, Shanxi University, Taiyuan, China; ^3^ School of Ecology and Environment, Anhui Normal University, Wuhu, China; ^4^ Phytotoxicity Research Department, Central Agricultural Pesticide Laboratory, Agricultural Research Center, Giza, Egypt

**Keywords:** RNAi, phloem-sucking pests, behavioral regulation, sugar receptor, sucrose

## Abstract

RNA interference (RNAi) is powerful biotechnology for studying the *in vivo* functions of key genes. Based on this property, RNAi can also be used for pest control as an effective alternative to chemical pesticides. The management of phloem-sucking pests is a tricky issue in current agricultural and forestry pest control. RNAi can silence key chemoreceptor genes of phloem-sucking pests; thereby regulating the behavior of these pests can be manipulated. So, it is considered to be a promising new type of ecological pest management strategy. In this study, we identified a candidate taste receptor gene, *BtGR11*, that controls the taste sensitivity to sucrose in the whitefly *Bemisia tabaci*, which is a serious invasive phloem-sucking pest worldwide. Functional analyses using the *Xenopus* oocyte expression system and the two-electrode voltage-clamp system revealed that the oocytes expressing BtGR11 responded to sucrose. Furthermore, we found that silencing *BtGR11* by RNAi inhibited the function of sensing sucrose in the whitefly. This study reports a key chemoreceptor gene that can be used for the understanding of the gustatory sensing mechanisms of whitefly to deterrent.

## 1 Introduction

RNA interference (RNAi) is a technology that involves post-transcriptional gene regulation, and it is a powerful tool for understanding various aspects of insect physiology (Saleh et al., 2006). Using RNAi for pest management requires the introduction of dsRNA *in vivo* to knockdown genetic targets of interest, thereby activating the siRNA pathway. By silencing specific genes, the roles of multiple functional genes in the sensing system have been elucidated (Huvenne and Smagghe, 2010; Zhao et al., 2011; Guan et al., 2021; Zhan et al., 2021). RNAi can also be used for pest control, as an effective alternative to chemical pesticides. For the future, RNAi is a promising ecological pest management strategy for phloem-sucking pests, which is a tricky issue in current agricultural and forestry pest control. Ac.

The interaction between phloem-sucking pests and their host plants involves many aspects (Li et al., 2019b; Jiang et al., 2019; Xia et al., 2021). In insects, the taste system not only has a significant impact on food selection, feeding, mating, and oviposition behavior (Isono and Morita, 2010), but it also has key functions in development and metabolism. Taste receptors play a vital role in insects that confer their ability to detect various chemicals and to use this information to make feeding decisions. Taste sensations from the environment are identified and evaluated by multiple gustatory receptors (GRs) and gustatory receptor neurons that are distributed in different tissues, including the antennae, proboscises, maxillary palps, labial palps, tarsi, wings, and ovipositors (Scott et al., 2001; Amrein and Thorne, 2005; Montell, 2009). Most receptors have four taste neurons and one mechanosensory neuron. In the taste neurons, one is a “sugar” neuron that is sensitive to sugars such as sucrose, glucose, and fructose; one is a “salt” neuron that is sensitive to salt; one is a “bitter” neuron that is sensitive to deterrent compounds such as quinine, chloroquine, caffeine, and strychnine; and one is a “CO_2_” neuron that is CO_2_ sensitive (Jones et al., 2007; Isono and Morita, 2010; Poudel et al., 2015; Poudel et al., 2017).

Previous studies of genomes and transcriptome analyses in insects such as *Drosophila melanogaster*, *Nilaparvata lugens*, *Anopheles gambiae*, and *Linepithema humile* have shown that the number of GR genes in a given species ranges from 16 to 96 (Hill et al., 2002; Robertson and Wanner, 2006; Wanner and Robertson, 2008; Smadja et al., 2009; Scott, 2018). To date, most of the studies related to gustatory receptors have been conducted on the model organism *Drosophila melanogaster.* In *Drosophila melanogaster*, GR43a (Miyamoto et al., 2012) and GR64f and GR5a (Jiao et al., 2008) respond specifically to D-fructose and trehalose, respectively, while the combination of GR64f and GR64a responds to multiple sugar components (Chyb et al., 2003; Jiao et al., 2007). In the silkworm, *Bombyx mori*, some gustatory receptors have the function of sensing fructose and inositol (Sato et al., 2011). In Helicoverpa armigera, several gustatory receptors exhibit sensitivity to galactose, maltose, sucrose, and fructose (Xu et al., 2012; Jiang et al., 2015). In Spodoptera litura, SlitGR8 has a specific response to D-fructose (Liu et al., 2019). In the brown planthopper, Nilaparvata lugens, fructose, galactose, and arabinose are ligands of *NlGR11*. The fecundity of brown planthoppers was affected after the expression of the *NlGR11* gene was inhibited (Chen et al., 2019).

The whitefly *Bemisia tabaci* (Gennadius) Hemiptera: Aleyrodidae) is one of the main pests in tropical and subtropical regions. In recent years, with global warming and frequent economic and trade exchanges between countries, the whitefly has also spread and invaded on a large scale. It is widely distributed in China, India, and other countries throughout the world, and has become a disaster pest worldwide (Chen et al., 2016). In China, there are currently two main biotypes of *B. tabaci*, B and Q. The list of host plants of the whitefly is extensive, including more than 600 plant species. Although there are many kinds of hosts, there are differences in the feeding and oviposition preferences for different host plants (De Barro et al., 2011; Tian et al., 2021). The success of the whitefly, in colonization, spreading, and even outbreaks in invasive areas is inseparable from its strong host adaptability (Wang et al., 2017). The excessive usage of pesticides is an important factor restricting the quality and safety of vegetables, such as tomatoes and peppers which is related to human health. Thus, explore new type of control technologies for whitefly is required. RNAi technology is a new, effective and promising pest control technology. At present, the control of the whitefly is heavily dependent on chemical pesticides, and there is a lack of effective green pest management technology to date. Develop RNAi techniques will provide technical support for the prevention and management of this insect. The taste sensing system of the whitefly plays an important role in its identification of and adaptation to different host plants. Understanding the taste mechanism can provide a theoretical basis for revealing the adaptive mechanism of the whitefly to host plants and understanding the invasion and rapid expansion mechanism of this invasive species. It will also provide a new potential target gene for whitefly management. At present, our understanding of the host plant adaptation mechanism of the whitefly mainly focuses on the olfactory system (Li et al., 2019a; Li et al., 2021; Zhan et al., 2021; Tian et al., 2022), so our understanding of the taste recognition of host plants by the whitefly is very lacking.

In the current study, we identified and cloned a candidate gustatory receptor gene, *BtGR11*, based on whitefly biotype B’s genome and transcriptome data. The ligands of *BtGR11* were identified using the *Xenopus* oocyte expression system and two-electrode voltage-clamp recording. We further verified the function of *BtGR11* by RNAi and insect behavioral techniques. The identification and functional study of this gene lay an essential foundation for understanding the whitefly’s mechanism of recognition and adaptation to host plants, and will allow for the development of techniques for the behavioral regulation of this insect.

## 2 Materials and methods

### 2.1 Insect rearing

The B biotype of whitefly (*Bemisia tabaci*) was reared in a greenhouse of the Beijing Academy of Agriculture and Forestry Sciences (Beijing, China), and identified as biotype B by the mitochondrial cytochrome oxidase I (mtCOI) gene. The whitefly population was raised on *Phaseolus vulgaris* plants in a cage (60 cm × 60 cm × 60 cm) with a 16 h day/8 h night photoperiod, a room temperature of 26°C ± 1°C, and a relative humidity of 65% ± 5%.

### 2.2 Cloning and sequence analysis of the *BtGR11* gene

RNA sample from different tissues of the whitefly was extracted using the Trizol Kit (Invitrogen, Dalian, China) according to the manufacturer’s instructions. RNA concentration and quality were detected using NanoDrop 2000 (NanoDrop Products, United States) and agarose gel electrophoresis. The extracted RNA was converted into cDNA using the PrimeScript RT reagent Kit with gDNA Eraser (perfect Real Time) (TaKaRa, Japan). The cDNA was stored at −20°C until the gene cloning and real-time PCR experiments.

Based on the genome and previously published transcriptome sequences (SRA numbers are SRX022878, SRA036954 and SRR835757) of the whitefly biotype B (Chen et al., 2016; Tian et al., 2021), one gustatory receptor gene with a complete open reading frame was identified. PCR primers were designed to clone the coding sequences of the BtGR11 gene based on the above predicted sequence (Table 1). Then, 2× TransStart FastPfu Fly PCR SuperMix (TransGen Biotech, China) was used to amplify the full-length coding sequences of the BtGR11 gene. The PCR product was purified using the MiniBEST DNA Fragment Purification Kit (TaKaRa, China), and the purified product was incorporated into the pEASY-Blunt Cloning vector, and then sequenced by TsingKe (Beijing, China). The open reading frame of BtGR11 was predicted using the online tool of ORF Finder (https://www.ncbi.nlm.nih.gov/orffinder/). Multiple sequence alignment was conducted using BLASTp (https://blast.ncbi.nlm. nih.gov/Blast.cgi) and DNAMAN. The transmembrane domains were predicted using TOPO2 (http://www.sacs.ucsf.edu/cgi-bin/open-topo2.py). The phylogenetic tree was built using MEGA-X (Kumar et al., 2018).

**TABLE 1:** List of primers designed and annealing temperatures used in this study.

Name	Primer sequence (5′-3′)	Length	Annealing temperature (°C)	Efficiency (%)
BtGR11 (F)	ATGGGCCCCAGCTCGAAA	1,242 bp	56	
BtGR11 (R)	AGG​GCA​GAT​TAT​AGA​TGA​GTT​TTT​AG			
BtGR11 RNAi(F)	taa​tac​gac​tca​cta​tag​ggC​AAT​TGG​AAC​AGC​CTA​CCG​T	498 bp	60	
BtGR11 RNAi(R)	taa​tac​gac​tca​cta​tag​ggA​CAT​CCA​TGA​AGT​TCC​AGG​C			
BtGR11 qPCR (F)	GAG​AGA​CGT​ACA​ACA​CCT​TGT​C	103 bp	60	111.17
BtGR11 qPCR (R)	GAG​CTG​CAG​GCA​AAT​GAA​ATA​A			
BtGR11 pT7Ts (F)	GCC​ACC​ATG​GGC​CCC​AGC​TCG​AAA​CAC​CT	1,242 bp	56	
BtGR11 pT7Ts (R)	AGG​GCA​GAT​TAT​AGA​TGA​GTT​T			
Actin qRCR (F)	AGA​GAG​AGG​ACA​GCT​TGG​ATA​G	92 bp	60	95.28
Actin qRCR (R)	CCC​AAG​GCC​AAC​AGA​GAA​A			
dsEGFP (F)	taa​tac​gac​tca​cta​tag​ggG​ACG​TAA​ACG​GCC​ACA​AGT​T	476 bp	60	
dsEGFP (R)	taa​tac​gac​tca​cta​tag​ggT​GTT​CTG​CTG​GTA​GTG​GTC​G			

### 2.3 Tissue expression profile of the *BtGR11* gene

The newly emerged whitefly adults (males and females) were dissected separately into head with antennae, head without antennae, thorax and abdomen for RNA extraction and synthesis of the cDNA. qRT-PCR was preformed using the qPCR Master Mix A6002 (Promega, China) system containing 7.2 μl of ddH_2_O, 10 μl of 2×GoTaq^®^ qPCR Master Mix (Promega, China), 10 μM of each specific primer, 100 ng cDNA. The qPCR program included 95 °C for 10 min followed by 40 cycles of 95°C for 15 s, 60°C for 1min β-actin was used as endogenous control. The amplification of BtGR11 and β-actin by qPCR was confirmed by 1% agarose gel electrophoresis. For standard curve, cDNA was used as template with five 10-fold different concentrations serial dilutions. The amplified fragments were sequenced to confirm the targeted gene sequences. The primer sequences for BtGR11 qRT-PCR are listed in Table 1. Each qRT-PCR experiment was performed with three biological replicates and three technical replicates.

### 2.4 Functional analysis of BtGR11 using a *Xenopus* oocyte ectopic expression system

The full-length sequence of BtGR11 was ligated into a pT7Ts expression vector using the ClonExpress II One Step Cloning Kit (Vazyme Biotech Co., Ltd., China) with the specific primers listed in Table 1. The constructed pT7TS vectors were linearized by restriction enzyme Smal I, and the cRNA of BtGR11 was synthesized using the Ambion mMESSAGE mMACHINE T7 Ultra Kit (Ambion, Austin, TX). Mature healthy *Xenopus* oocytes were treated in washing buffer (5 mM MgCl_2_, 2 mM KCl, 96 mM NaCl, and 5 mM HEPES [pH = 7.6]) with 2 mg/ml collagenase I for 1 h at room temperature until almost all of them were separated. After culturing overnight in an 18°C incubator, 50 ng BtGR11 cRNA was microinjected into each oocyte.

The injected oocytes were cultured in an incubation medium (1 × Ringer’s buffer, 5% dialyzed horse serum, 50 mg/ml tetracycline, 100 mg/ml streptomycin, and 550 mg/ml sodium pyruvate) at 18°C for about 3 days. Then, the reaction of the cultured oocytes to the ligands was tested using a two-electrode voltage clamp system (OC-725C oocyte clamp, Warner Instruments, Hamden, CT, United States). A total of eight sugars were tested in the experiment, including D-fructose, D-glucose, D-sucrose, D-galactose, D-arabinose, D-maltose, D-mannose, and D-xylose (Supplementary Table S1). These compounds were purchased from Sigma and had a purity of ≥99.5%. The sugars to be measured were dissolved in water to a concentration of 0.1 M. Then, the solution was diluted to 10^−4^ mol/L using 1× Ringer solution (2 mM KCl, 96 mM NaCl, 5 mM MgCl_2_, 5 mM HEPES, and 0.8 mM CaCl_2_, pH 7.6). The data were recorded and analyzed using Digidata 1440 A and PCLAMP 10.2 software (Axon Instruments Inc., Union City, CA, United States).

### 2.5 Functional validation of *BtGR11* by RNAi

**FIGURE 1:**
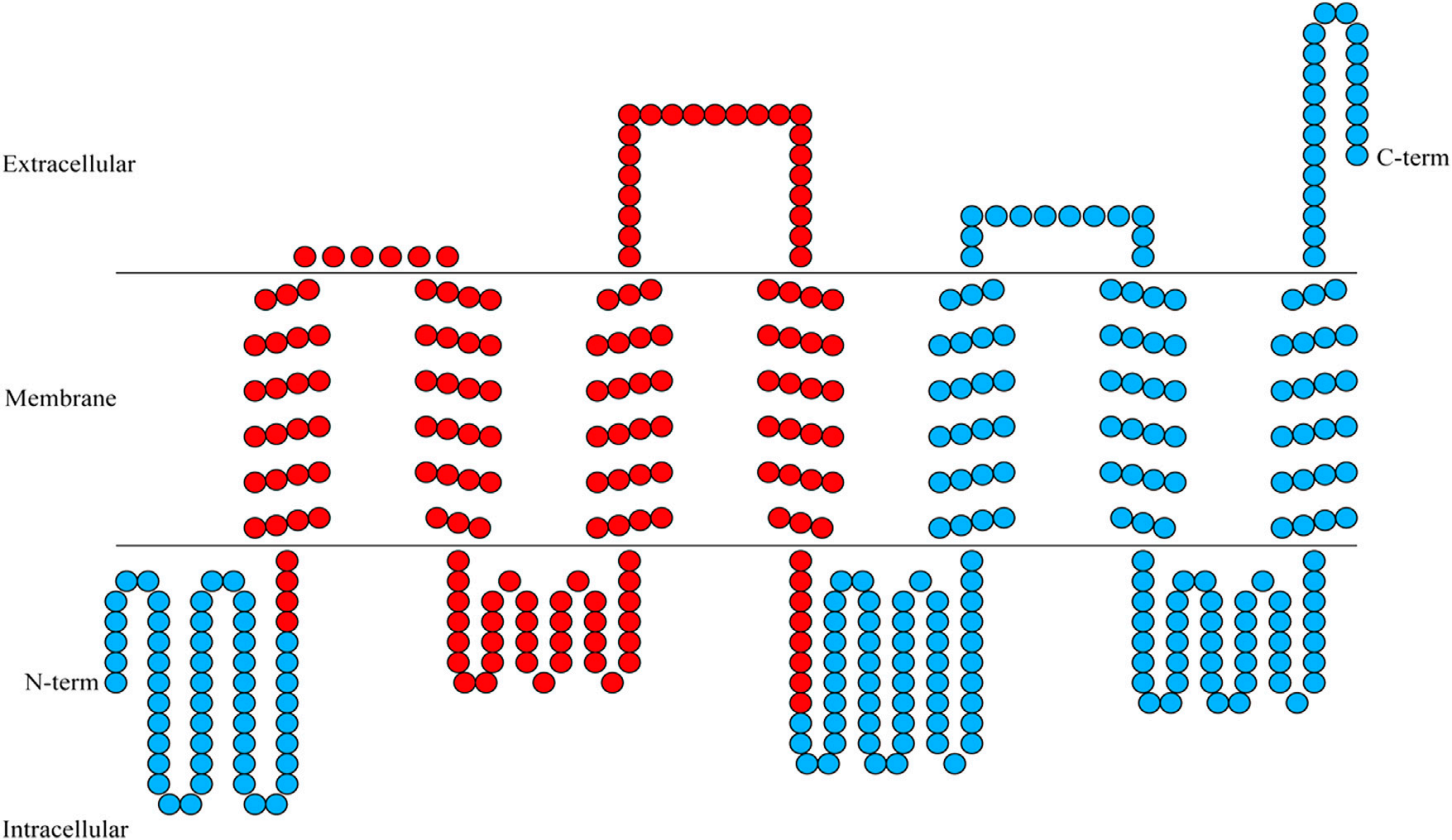
Transmembrane helical structures in the amino acid sequence encoding BtGR11. Each circle represents an amino acid, and the red circles represent the region of RNAi.

RNA interference of the whitefly was carried out according to our previously reported method (Li et al., 2021). The dsRNA primers were designed using the E-RNAi web tool (https://www.dkfz.de/signaling/e-rnai3/) (Table 1). The red circle in Figure 1 represents the region of RNAi. The dsRNAs were amplified using primers in Table 1 from the GR11 plasmid Cloning vector cloned into pEASY-Blunt vector. dsRNA was obtained using the TranscriptAid T7 High Yield Transcription Kit (Thermo Fisher Scientific, DE, United States) according to the manufacturer’s instructions. The synthesized dsRNA of BtGR11 and EGFP (Enhanced Green Fluorescent Protein) was diluted up to 500 ng/μl with a 30% glucose water solution. The double-layer film feeding method was performed to interfere with the gene mRNA expression levels of the newly emerging whitefly using a female-to-male adult ratio of 1:1. The interference lasted for 2 days, and then the reduced expression of the mRNA level was evaluated by qRT-PCR. The primers of the qRT-PCR are listed in Table 1. The successfully disturbed whiteflies were chosen for subsequent behavioral and biological studies.

### 2.6 Behavioral and fecundity experiments

To test the ability of the whiteflies to detect sucrose, adults were deprived of food for 24 h (only supplied with water). Then, 50–60 adult whiteflies (1:1 female/male ratio) were selected and placed into a glass tube (45 cm length ×3 cm diameter) with a double-layer film at both ends of the tube. The double-layer film at one end contained a sucrose solution colored with red dye (0.2 mg/ml sulforhodamine B, Solarbio,Lot.No.922E021) and the film at the other end contained distilled water colored with blue dye (0.125 mg/ml brilliant blue FCF, Solarbio, Lot.No.917E031). The test was conducted in an appropriate environment conditions of 25 ± 1°C, 75 ± 5% relative humidity and under dark conditions. After 12 h, the abdomen colors of whiteflies were observed using a stereomicroscope. Whiteflies that fed on sucrose had a red color in their abdomens, while whiteflies that fed on distilled water had a green color in their abdomens, and whiteflies that fed on both solutions had a purple color in their abdomens. The preference index (PI) was calculated with the following formula: PI = (N Red +0.5 N Mix)/(N Red + N Blue + N Mix) (Weiss et al., 2011), where N Red is the number of whiteflies with the red color, N Blue is the number of whiteflies with the green color (resulting from the blue dye) and N Mix is the number of whiteflies with the purple color (Figure 5A). The PI is considered to reflect a preference when the value range is between 0.5 and 1, or no preference when the value is below 0.5. The attractiveness was tested using D-fructose with a range of concentrations (0.05 M, 0.10 M, 0.30 M, 0.50 M, 0.80 M, and 1.0 M). The sucrose recognition preference before and after BtGR11 gene silencing was also evaluated by the above method. All experiments were replicated three times.

For the whitefly fecundity experiment, the female adults were transferred to three-week-old common bean plants after 48 h of feeding on dsRNA. Each group of five whiteflies was placed in a clip cage, and the number of eggs in each cage was recorded after 3 days. The plants and whiteflies were maintained in a plant growth chamber under a photoperiod of 16 h light: 8 h dark, at 25°C and 65% relative humidity.

### 2.7 Statistical analysis

The relative gene expression level was calculated using the relative standard curve method with the help of the QuantStudio 7 Flex Real-Time PCR system (ThermoFisher, United States). The mRNA relative expression levels were analyzed by the 2^−ΔΔCT^ method. and the statistical significance was conducted using the IBM SPSS Statistics 22.0 statistical analysis system.

## 3 Results

### 3.1 Cloning and sequence characterization of *BtGR11*

According to the sequencing results, the ORF of BtGR11 (Genbank ID OP653787) is 1,242 bp (414 amino acids). Transmembrane structure analysis showed that the BtGR11 protein contains seven putative transmembrane domains, located in TM-I = 56–78 amino acids (aa); TM-II = 85–107 aa; TM-III = 142–164 aa; TM-IV = 188–210 aa; TM-V = 271–293 aa; TM-VI = 306–328 aa; and TM-VII = 371–393 aa (Figure 1).

**FIGURE 2:**
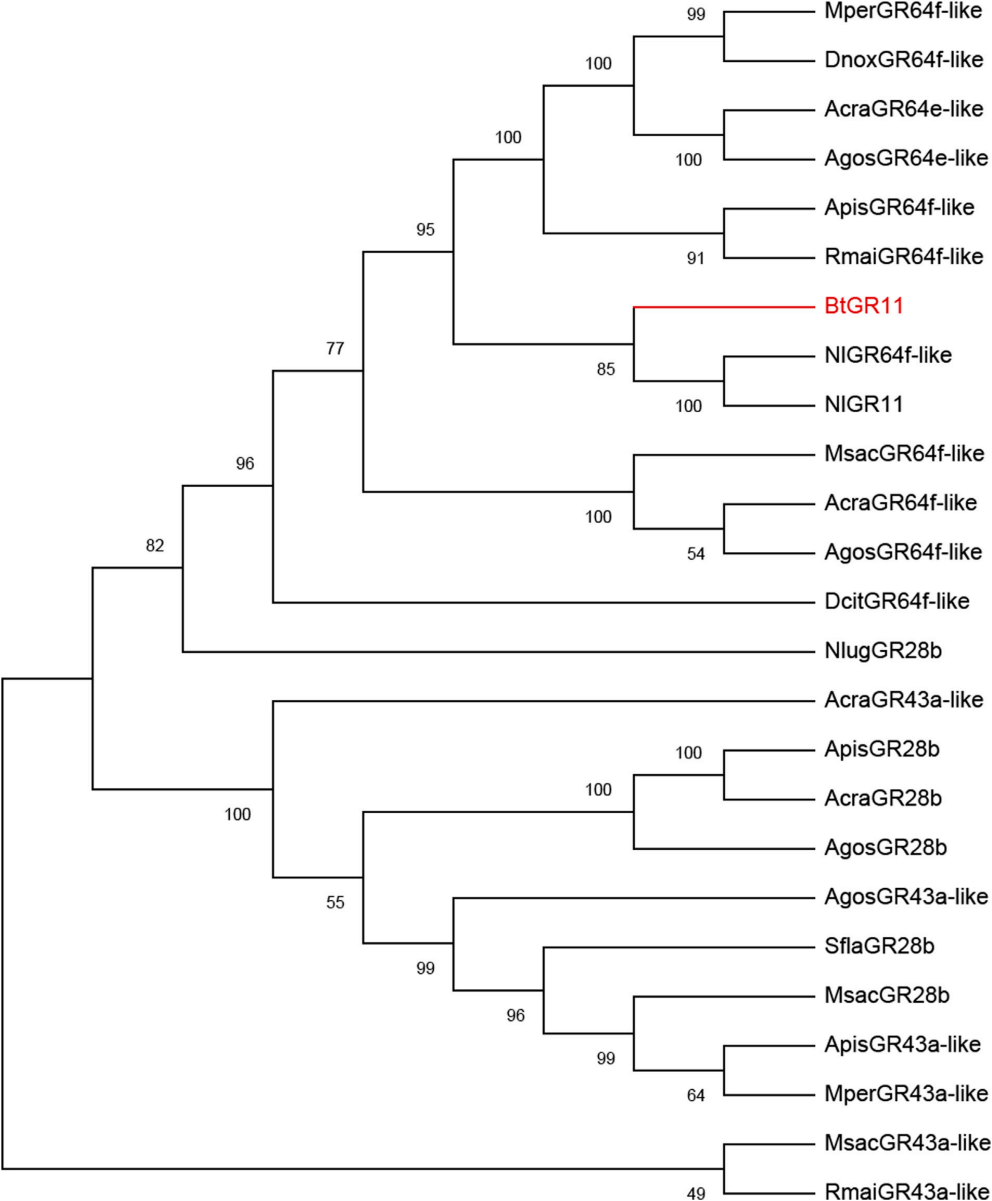
Phylogenetic tree of BtGR11 and other hemipteran gustatory receptors based on amino acid sequences. Gene names and GenBank numbers are as follows: ApisGR28b, ApisGR43a-like, ApisGR64f-like: *Acyrthosiphon pisum* (XP_008178128.1, XP_003244120.4, XP_001942787.2); AcraGR43a-like, AcraGR64e-like, AcraGR64f-like, AcraGR28b: *Aphis craccivora* (KAF0773920.1, KAF0765871.1, KAF0765661.1, KAF0749339.1); AgosGR28b, AgosGR43a-like, AgosGR64f-like, AgosGR64e-like: *Aphis gossypii* (XP_027840408.1, XP_027840395.2, XP_027839680.2, XP_027847461.1); DcitGR64f-like: *Diaphorina citri* (XP_017300309.2); DnoxGR64f-like: *Diuraphis noxia* (XP_015369418.1); MperGR43a-like, MperGR64f-like: *Myzus persicae* (XP_022165389.1, XP_022180782.1); MsacGR43a-like, MsacGR64f-like, MsacGR28b: *Melanaphis sacchari* (XP_025195577.1, XP_025204513.1, XP_025202779.1); NlugGR28b, NlugGR11, NlugGR64f-like: *Nilaparvata lugens* (XP_039287957.1, AUD08731.1, XP_022186608.2); RmaiGR43a-like, RmaiGR64f-like: *Rhopalosiphum maidis* (XP_026807230.1, XP_026822156.1); SflaGR28b: *Sipha flava* (XP_025417386.1)

The N-terminus of the BtGR11 protein is located in the cell membrane, and the C-terminus is located outside the cell membrane. The amino acid sequence of BtGR11 was searched for homologous sequences in the NCBI database using BlastP, and the BtGR11 protein was found to be highly similar to the GR sequences of various hemipteran insects. The amino acid sequence similarities between BtGR11 and *Rhopalosiphum maidis* GR64f-like (XP_026821636.1), *Myzus persicae* GR64f-like (XP_022180784.1) and *Nilaparvata lugens* GR 11 (AUD08731.1) were 61.35%, 59.93% and 58.44%, respectively. The results of the protein multiple sequence alignment showed that similar sequences were mostly located in the second half of the protein sequences (Supplementary Figure S1). After building a phylogenetic tree with the amino acid sequences of BtGR11 and the other hemipteran GR genes, BtGR11 was found to cluster into the gustatory receptor GR64f-like subfamily (Figure 2).

### 3.2 Tissue expression profiles of BtGR11

**FIGURE 3:**
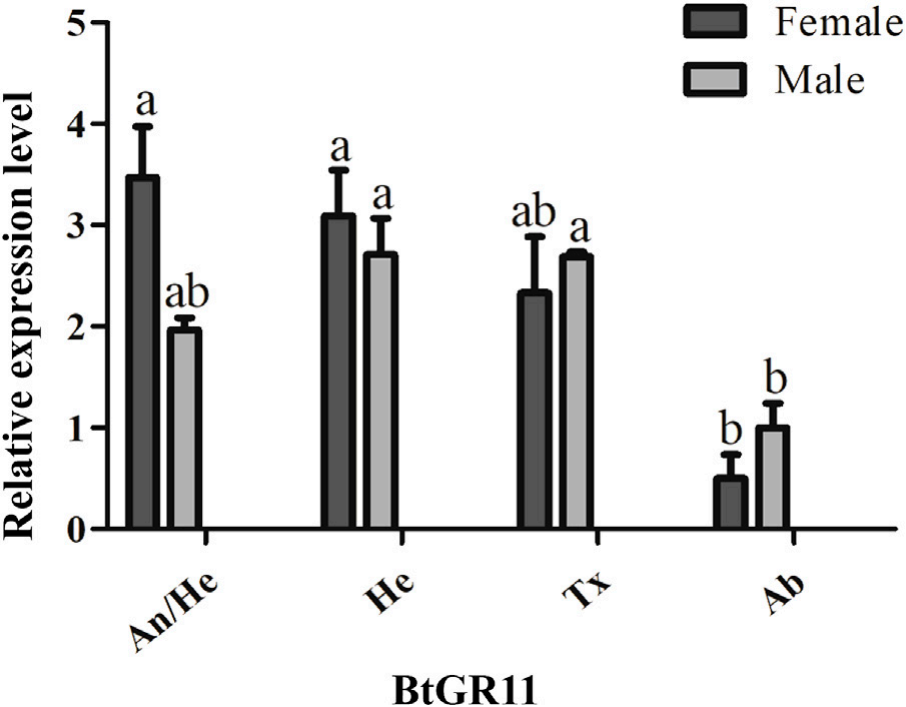
Tissue expression profiles of the BtGR11 gene. Female: female adult; Male: male adult; An/He: head with antennae; He: head without antennae; Tx: thorax; Ab: abdomen.

The mRNA relative expression levels were tested by qRT-PCR using different tissue samples. Standard curve plots with correlation coefficient (R^2^) values were above 0.99 (Supplementary Figure S2). This indicated the measurement of the relative expression level of the BtGR11 was accurate. The representative pictures of qRT-PCR efficiency checking is shown in Supplementary Figure S2. The qRT-PCR results indicated that the expression of the BtGR11 gene in whitefly female adults was highest in the head with antennae, and relatively high in the head without antennae and thorax. In male adults, the expression of the BtGR11 gene was highest in the head without antennae, with moderately high expression levels mainly in the thorax and head with antennae (Figure 3).

### 3.3 Functional assay of BtGR11 using two-electrode voltage-clamp recording

**FIGURE 4:**
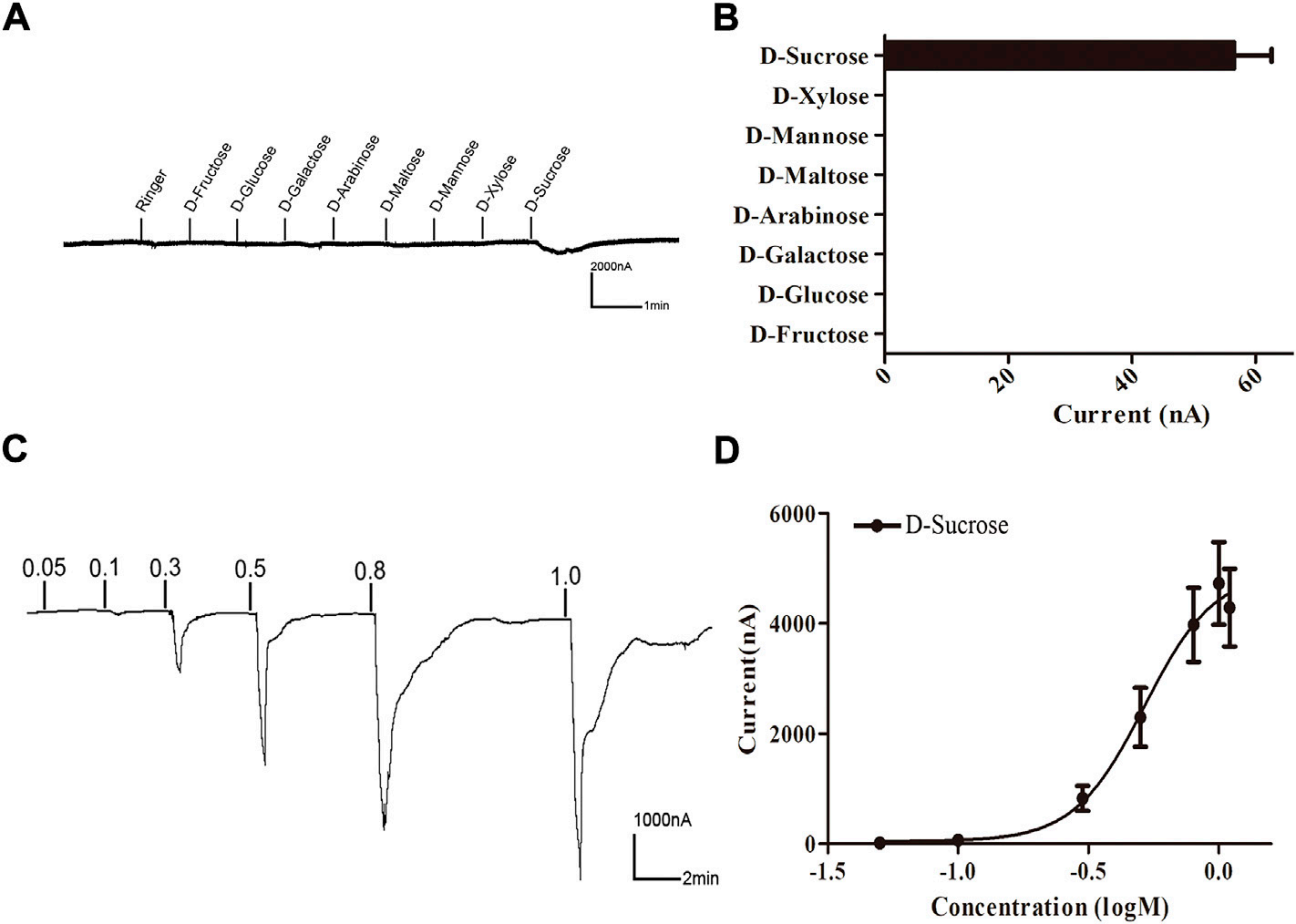
Responses of *Xenopus* oocytes expressing BtGR11 to different sugar ligands. **(A)** Inward current responses of *Xenopus* oocytes injected with BtGR11 cRNA to sugar compounds (0.1 M). **(B)** Response profile of *Xenopus* oocytes expressing BtGR11. Error bars indicate SEM (n = 4). **(C)** Response of BtGR11-expressing *Xenopus* oocytes to D-sucrose at different concentrations. **(D)** Dose–response curve of BtGR11-expressing *Xenopus* oocytes to D-sucrose, with EC_50_ = 0.563 M. Error bars indicate SEM (n = 4).

A *Xenopus* oocyte expression and voltage-clamp recording system was used to test the functionality of BtGR11. Eight sugar ligands were selected for testing. First, BtGR11 was tested using 0.1 M of each ligand to determine its responsiveness, and the results showed that BtGR11 was only responsive to sucrose (Figure 4). Then, a range of sucrose concentrations (0.05–1.1 M) was tested to determine the dose-response relationships. According to the dose-response curve, the sucrose-induced current increased with the concentration from 0.05 M to 1.1 M, and the EC_50_ value of sucrose was 0.563 M (Figure 4C,D).

### 3.4 Behavioral preferences of whitefly for sucrose

**FIGURE 5:**
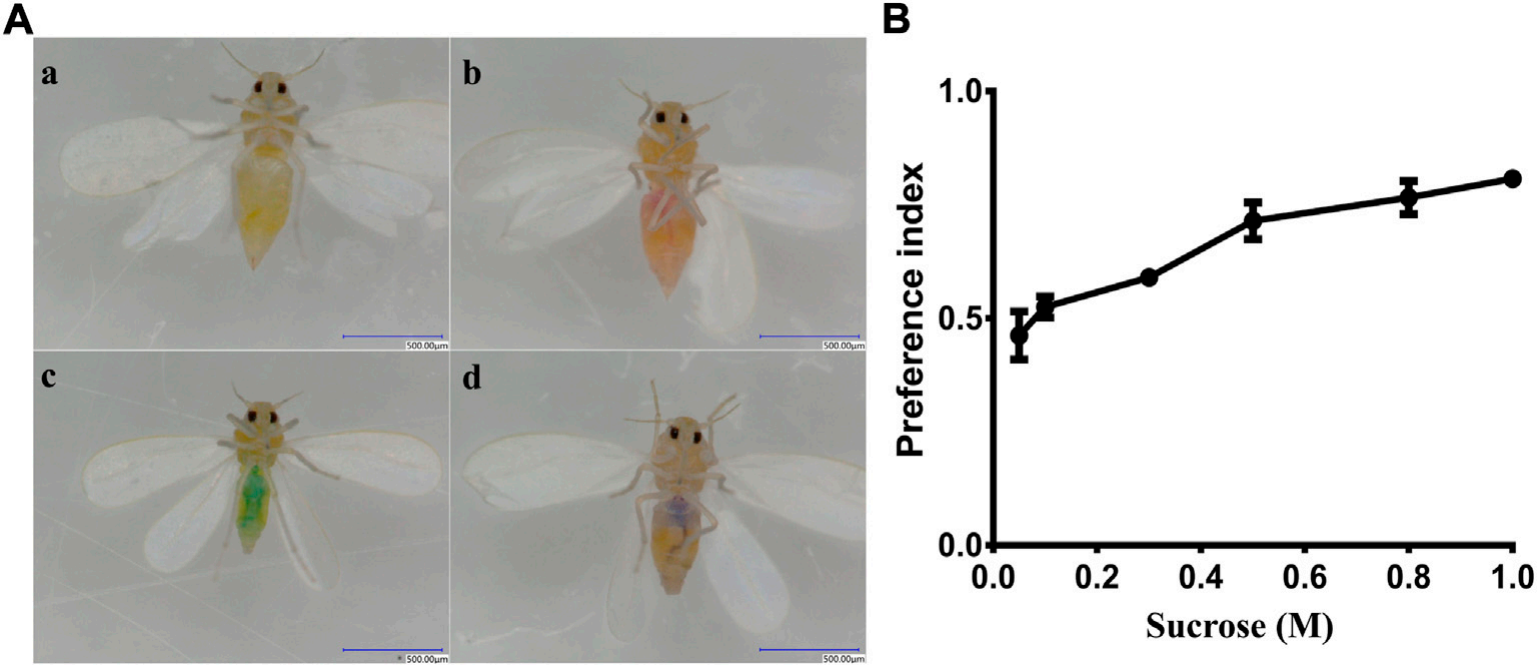
Behavioral preference of whitefly to sucrose. **(A)** **(a)** whitefly fed nothing; **(b)** whitefly fed sucrose (with red dye); **(c)** whitefly fed water (with blue dye); **(d)** whitefly fed both sucrose and water. **(B)** PI values for sucrose.

We conducted a two-way choice test to estimate the preference of whitefly for sucrose. The results indicated that whitefly adults showed a clear preference for sucrose compared with water (Figure 5A). The attraction preference index was increased with increasing sucrose concentrations (Figure 5B). The lowest PI of sucrose was 0.462 at a concentration of 0.05 M, while the highest PI was 0.807 at the 1 M concentration. These data suggest that whitefly adults have a preference for sucrose in a dose-dependent manner.

### 3.5 Interfering with BtGR11 affects sucrose recognition

**FIGURE 6:**
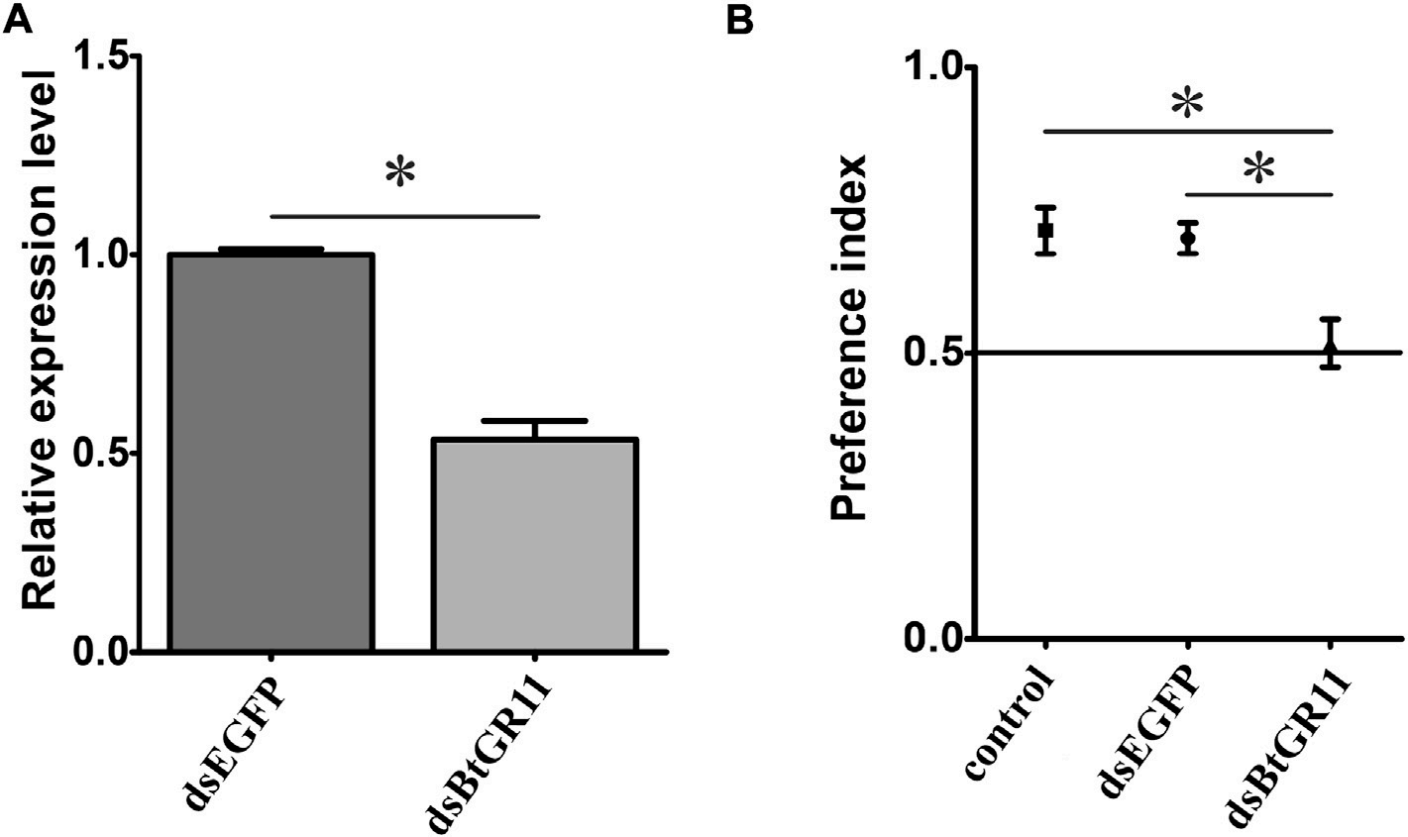
Preference index of female whitefly after interference of BtGR11. **(A)** the mRNA relative expression of the BtGR11 after interfering. **(B)** Preference index of female whitefly for sucrose, control: untreated group, dsEGFP mean EGFP control group, and dsBtGR11 mean BtGR11 RNAi experimental group. * mean *p* < 0.05.

After interfering with the BtGR11 mRNA gene expression, the relative expression of the BtGR11 gene significantly decreased by 47% after 48 h compared with the EGFP control group (student t-test, *p* < 0.05) (Figure 6A). After interference with dsBtGR11, the PI value decreased by 0.22 compared with the untreated group, and the PI value decreased by 0.18 compared with the EGFP control group. There was no difference in the preference of whitefly for sucrose between the untreated group and the EGFP control group, but there was a significant difference between the experimental group and these two (student t-test, *p* < 0.05, Figure 6B), indicating that the RNAi of the BtGR11 gene would affect the preference of whitefly adults for sucrose. We further evaluated the effect of BtGR11 interference on whitefly fecundity. After 48 h of interference with dsBtGR11 and dsEGFP, the average numbers of eggs laid by five female adults for 3 days were 149 and 152, respectively. These results showed that the disturbance of BtGR11 expression did not affect the fecundity of whitefly (Supplementary Figure S3).

## 4 Discussion

The gustatory system plays a crucial role in a variety of insect behaviors, including feeding, mating, and many others (Isono and Morita, 2010). Functional studies of GR genes are important for a better understanding of the mechanisms of gustatory perception, and may also provide potential targets for developing a better strategy for pest control technologies (Weiss et al., 2011). In this study, we cloned a gustatory receptor gene, BtGR11, based on the genome and transcriptome data of whitefly biotype B. BtGR11 has seven transmembrane domains, and the N-terminus of the protein is located in the cell membrane, while the C-terminus is located outside the cell membrane, which is consistent with the previously published structural characteristics of gustatory receptor proteins (Kang et al., 2018).

Through multiple sequence alignment and phylogenetic tree analysis, the BtGR11 gene was found to be in the same clade with the GR64f-like gustatory receptors of other insects, indicating that BtGR11 may belong to the GR64f-like subfamily and have similar functions. A study on GR64f-like in *D. melanogaster* showed that the DmelGR64f gene is broadly active as a coreceptor for detecting sucrose, maltose, and glucose (Jiao et al., 2008). Currently, there are few studies on the functions of GR64f-like in agricultural pests. One study of the GR64f gene (NlGR11) in *N. lugens* showed that NlGR11 can recognize fructose, galactose and arabinose, and disturbing NlGR11 affected the fecundity of *N. lugens* (Chen et al., 2019). The current study found that BtGR11 in whitefly is specifically sensing sucrose. This result is different from the previously reported functions of GR64f-like genes in insects, so the findings in this study expand our understanding of the functions of GR64f-like genes in insects.

Studying the expression of gustatory receptor genes in different tissues of insects is useful for speculating on the possible functions of the receptors (Miyamoto et al., 2012). It has been reported that the GR genes have a wide distribution, and they are distributed in various tissues of insects. Tissue expression profiling showed that the BtGR11 gene was highly expressed in the head and head with antenna. This may be due to the various taste organs, including mouthparts, in the head of whitefly adults. We noticed that BtGR11 was also expressed in the thorax and abdomen. The GR gene is reportedly not only expressed in the taste organs, but also in the antennae, thorax, abdomen, and reproductive organs. For example, the GR gene of *Heliconius melpomene* is mostly expressed in the labial palps, proboscis, antennae and legs of female and male adults, and the female-specific GR gene is mainly expressed in the legs (Briscoe et al., 2013). In *N. lugens*, the GR genes are distributed in adult head, leg, midgut, fat body and ovary (Kang et al., 2018). We speculate that whitefly BtGR11 not only acts as a carbohydrate sensor in chemosensory receptors, but is also used in other organs to assess nutrients. In *Drosophila* adults, GR43a is present in the brain and gut, which is sufficient to assess nutrient carbohydrates and regulate feeding behavior (Miyamoto et al., 2012). In the foregut of the cotton bollworm, HaGR9 acts as a nutrient sensor to guide the digestive process and protect against harmful substances (Xu et al., 2012).

Functional analysis using the *Xenopus* oocyte expression and voltage-clamp recording system showed that whitefly BtGR11 was specifically responsive to D-sucrose among the eight tested sugar compounds. Sucrose is the most common sugar translocated in plants. Large quantities of sucrose accumulate in the edible parts of some plants, making it the most abundant natural sweetener in food (Salvucci, 2003). A previous study using artificial diets containing radiolabeled carbohydrates showed that the whitefly rapidly hydrolyzes and metabolizes ingested sucrose (Salvucci et al., 1997). In addition to providing nutrients for the whitefly, when the carbon input from sucrose exceeds the metabolic needs, trehalose is synthesized from the excess sucrose for honeydew excretion (Salvucci, 2003). In this study, a series of *in vitro* and *in vivo* functional experiments showed that BtGR11 is required for the whitefly’s recognition of sucrose.

Sugar can stimulate feeding behavior in insects (Scott, 2018), most insects rely on sugars for their energy needs, and the insect lifespan and fecundity are limited by the energy ingested and expended (Wäckers, 2001). After the interference of BtGR11, the fecundity of the whitefly did not decrease significantly. There may be other sugar receptors involved in the effects of BtGR11 on fecundity in the whitefly. Although no difference in fecundity was detected after BtGR11 interference, its sensitivity to sucrose was significantly inhibited, which may have various effects on the insect’s lifespan, development and metabolism. These specific effects need to be further investigated in the future. In addition, studies have shown that various sugars of whitefly, including sucrose, have important effects on the detoxification of defensive metabolites in host plants. For example, the whitefly detoxifies the majority of ingested glucosinolates by the stereoselective addition of glucose moieties, which prevents the hydrolytic activation of these defense compounds (Malka et al., 2020). The whitefly has feeding and oviposition preferences for different plants, and even different varieties of the same kind of plant (Li et al., 2016; Li et al., 2021). The whitefly may sense the sucrose content in plants to judge whether they are suitable hosts for feeding and/or oviposition, because sucrose is often the main saccharide in the phloem vasculature, with concentrations ranging from 0.4 to 0.8 M (Rennie and Turgeon, 2009; Malka et al., 2020). Therefore, the identification of BtGR11 will help us to understand the mechanisms of whitefly selection and recognition among different host plants. In the future, by silencing the expression of GR11, the feeding and nutritional supplementation of the whitefly could be inhibited; thereby the host selection, nutrient acquisition and development of this pest can be manipulated, so as to achieve the purpose of controlling the whitefly population.

## 5 Conclusion

In conclusion, this study revealed the function of the BtGR11 gene in the whitefly. The full-length cDNA of BtGR11 was cloned. In addition, the tissue expression profiles of BtGR11 in male and female adults indicated that this gene may have specific taste functions in different tissues of both sexes. The specific sensitivity of BtGR11 to D-sucrose was determined using the *Xenopus* oocyte expression and voltage-clamp recording system. Furthermore, the effect of BtGR11 on sucrose recognition in whitefly was determined by RNAi. These results are crucial for improving the understanding of the role of taste in whitefly host recognition and adaptation, and also provide a potential target gene for the development of new control methods for whitefly.

## Data Availability

The sequence of BtGR11 presented in the study is deposited in the NCBI GenBank, accession number OP653787.
