# Supplementary material for: Silencing the gustatory receptor BtGR11 affects the sensing of sucrose in the whitefly Bemisia tabaci
Source: Front Bioeng Biotechnol. 2022 Nov 14;10:1054943. doi: 10.3389/fbioe.2022.1054943 (PMC9702514; doi:10.3389/fbioe.2022.1054943)
Supplement: Supplementary file 1 [file DataSheet1.pdf]

Table S1 Types of sugars used in this study

Number	Name	Molecular formula	CAS number	Chemical structure
1	D-fructose	C6H12O6	57-48-7	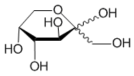
2	D-glucose	C6H12O6	50-99-7	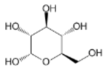
3	D-sucrose	C12H22O11	57-50-1	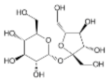
4	D-galactose	C6H12O6	59-23-4	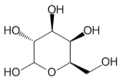
5	D-arabinose	C5H10O5	10323-20-3	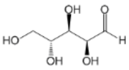
6	D-maltose	C12H22O11•H2O	6363-53-7	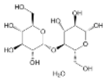
7	D-mannose	C6H12O6	3458-28-4	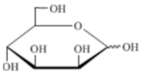
8	D-xylose	C5H10O5	58-86-6	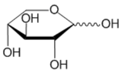

```
BtabGR11         .................................................MGPSSKHLTLENFPSHPDSLHRALKPVLILARCFALLPVEGISAPNASYLA    51
RmaiGR64f-like   ..............................................MLHDN...TEPVYLKRRDFAESAYGQQQPQPS.ALQLADSAFNSKDNTEKNT.K    49
MperGR64f-like   ..............................................MSDDDSNNTGPVYLKHRNFAD...GRQQPQPTSASHLVDSAFNSKFNKGKNISK    51
NlugGR64f-like   MSMFNKNPRNVQDVSRRLNQVMGPKITEDHHSAHLLLANTPPDGGYVLDIDRHDYDADLNKGPHTPDELHVALRPVLVLAQCFALMPLYGVSSPHPKHLR   100
AcraGR64f-like   .............................MSAVHDIRRNKHNSVSPSMLSAQEYSAREFNEYLNKTG.VHYYIRYVLTFASIFGVFPVSSIYRRDVYKLT    70
Consensus                                                                                        a

BtabGR11         FNWNSLPCIYLGLVIGYSVLIALLSIWKTVQ.........FGISYYSTVDFVFVTSSMVIYLLFLQLATLWPKTMCQWEKAEHG.MRHYGYPKR......   135
RmaiGR64f-like   LEIQDELGMPVNYVRIKALEDDIRMNIISDE.........NSLHRAITPVLILAQICALLPVQGIL.GKNTSYLVFNWFSCTVIYVFIVIAASL......   133
MperGR64f-like   LEIQDDLGMPVNYVRIKALEDDIRMNIISDK.........NSLHRAITPVLILAQICALLPVQGIR.GQNTSYLVFNWFSWPVIYVFVVIAASL......   135
NlugGR64f-like   FQWFSIRVLYTCALVVGSITIVILSIIRTVN.........NGISFLSTVDFVFFGSSAIVALLFLKLATEWKQLVDEWGPAERKIIRIYGPLKK......   185
AcraGR64f-like   FRWYYPIAIYSFLLFCGFASIELFSLDYTIRNLNQDNLTAKGGIKKATSGSIFYGNACCALWMFIKLAKKWPKLMQDWRTVEIS.MRRFGAPRLGWKSTT   169
Consensus                                                       t                             w

BtabGR11         MALKFRFILFVMMALAI...VEHVASIVTAVFEALPCSSGGW..DIVRAYFVTKFRQVFTFVP..........FNICIALPVLVMNLILTCAWNFMDVFI   220
RmaiGR64f-like   LILSFSLIKIYMTGLTYYSTVEHSASVLTGIMKAIPCSTGGL..DIFRAYFSMSFRQVFTLIN..........YSLVIAIPLGLFNFMLACAWNYMDLFI   221
MperGR64f-like   LILSFSLIKIYMTGLTYYSTVEHSASVITGIMKAIPCSTGGL..DIFRAYFSMSFRQVFALIH..........YSLVIAIPLGFLNLMLACAWNYMDLFI   223
NlugGR64f-like   LPNQMRTMAYTVMALAF...AEHVTATATHVANALPCTGGEWNWRLAEAYYHFAFRQVFSQTE..........VSLWIAAPLSVLNLLVTCAWNFMDLFI   272
AcraGR64f-like   LAIVLLVFAFTEHGLHNWLNTRPGGKDDIASMALIDNDDNGSLILLDQSATFTGYLERFSLKTHWYLYDDYDSYTPVKGFLVMWLSLTATFLWNFTDLFI   269
Consensus                      l                                           f                                 wn  d fi

BtabGR11         ILMSFAMAERYKQVNERLNSVKCKVLPTSFWRHIRETYNTLSCLTKLLDNLLSPIVLLSFANNLYFICLQLLNSLRPMQS.TWQATYFVYSFSYLLVRTI   319
RmaiGR64f-like   IILSSALADKFKQLNQKLASVKGKVLPSTYWRKSRETYNLLASLTKDFDEFLSPVILLSFANNLYFICLQLLNSLKPMHD.VWEAIYFVFSFTYLVGRTC   320
MperGR64f-like   IILSCALSDKFKQLNQKLATVKGKVLPSTYWRKSRETYNLLASLTQDFDEFLSPVILLSFANNLYFICLQLLNSLKPMHN.VWEAIYFVFSFTYLVGRTC   322
NlugGR64f-like   MVLSVALAARFKQINDKLESVKGKVMPRWFWRQTRESYNTLSCLTKRLDETLSLIVLLSFANNLYFICLQLLNSLKPLRN.LWQAIYFVFSFGYLVLRTA   371
AcraGR64f-like   MLVSSALAAQFKKLTKAMHGVRGQMLTMSQWQEYRETYTSLTHLVKKIDDHINVIVALSIGSNIYFICAQLITEIDSIKHSYFRTLFYMYSFMFLVFRTT   369
Consensus           s a     k        v         w   re y  l  l    d        ls   n yfic ql                   sf  l  rt

BtabGR11         AVSLFAASVHDQSKEPKSVLFSVPTESYCVEVSRFLIQVTNDDLALTGCKFFAVTRTFMLTVAGTIVTYEIVLVQFNAVSSEDAAAAKNSSIIC         413
RmaiGR64f-like   AVSLYAASINDQSKKPKAILFSVPTESYGVEVARFLMQVTSDELALTGCNFFSVTRTLMLTVAGTIVTYEIVLIQFNSVNS.EVNDQNNTIIYC         413
MperGR64f-like   AVSLYAASINDQSKKPKAILFSVPTESYGVEVARFLTQVTSDELALTGCNFFSVTRTLMLTVAGTIVTYEIVLIQFNSVNS.DGNGQNNTTTYC         415
NlugGR64f-like   TVSLFAAHIFDESKRPVSVLFSVPSECYCTEVQRFLTQVTSDNLALTGCRFFSVTRTLMLTVSIPSINSNQ.......................         442
AcraGR64f-like   AVVMQASAINDESKKIVPEIFMCPTHSYSIETQRFLQEVTSDYVALTGLKMFSITRNFLLGVAGAVLTYEIVLIQLQNTN..............         449
Consensus         v   a    d sk      f  p   y  e  rfl  vt d  altg   f  tr   l v
```

Fig S1. RmaiGR64f-like gene sequences: Rhopalosiphum maidis (XP_026822156.1),MperGR64f-like: Myzus persicae (XP_022180782.1),NlugGR64f-like: Nilaparvata lugens (XP_022186608.2),AcraGR64e-like: Aphis craccivora (KAF0765871.1). Consensus: Consensus sequence. Black areas represent 100% similarity, pink areas represent more than 75% similarity, and light blue areas represent more than 50% similarity. The Latin name for are not italicized.

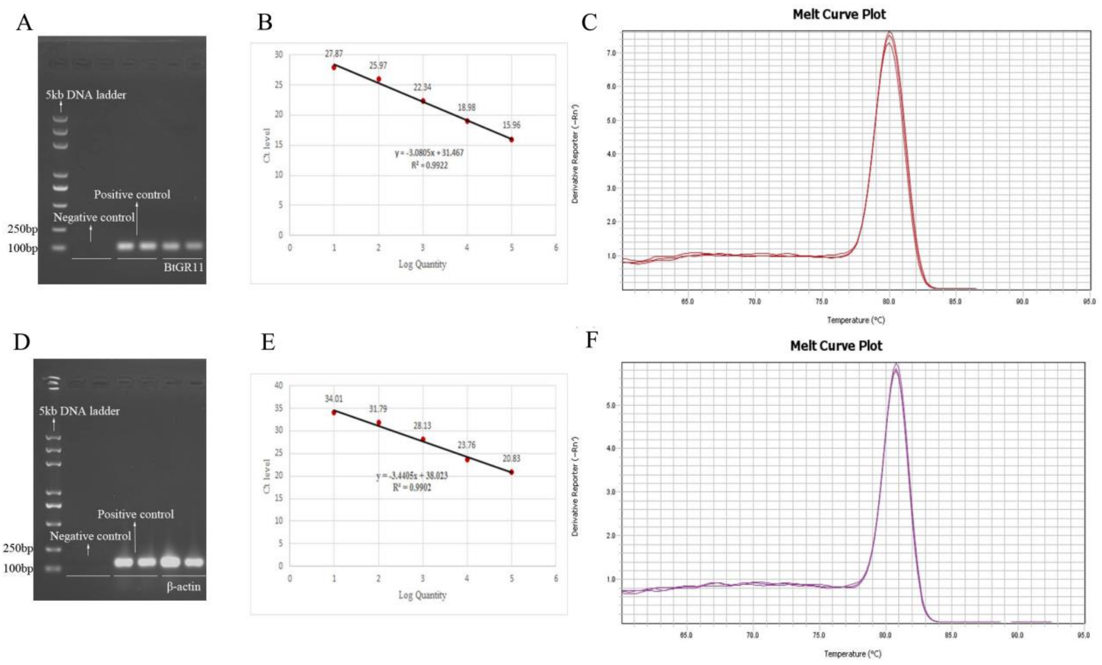

Fig S2. Representative results of qPCR analysis. (A) qPCR product gel electrophoresis for BtGR11 on 1% agarose gel, (B) standard curve for BtGR11 with R^2 = 0.992, (C) Melt curve for BtGR11 standard cDNA amplification, (D) qPCR product gel electrophoresis for β-actin on 1% agarose gel, (E) standard curve for β-actin with R^2 = 0.990, (F) Melt curve for β-actin standard cDNA amplification.

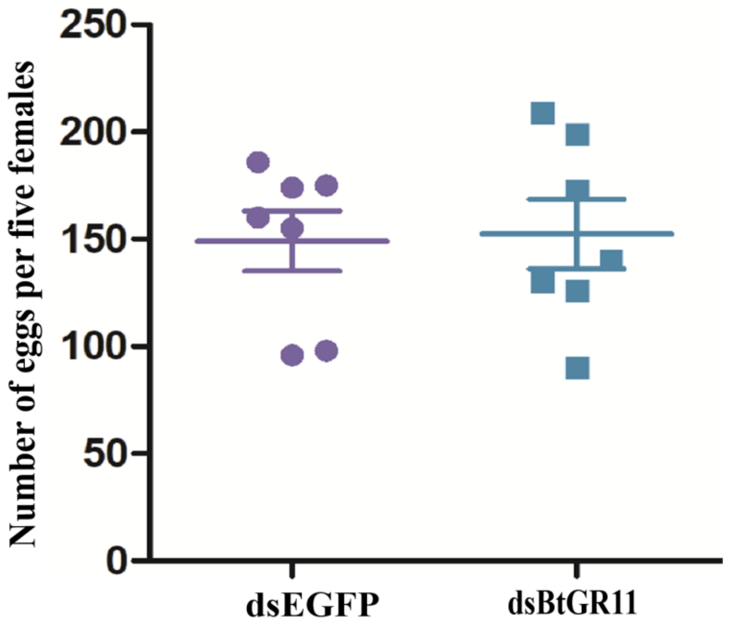

FigS3. Numbers of eggs laid by female whiteflies after interference of BtGR1
